# Supplementary material for: Exploring cardiovascular disease prevalence and contributing factors among adults in Southern Iran, a cross-sectional survey: Rationale, design, and primary results
Source: PLoS One. 2025 Nov 18;20(11):e0335199. doi: 10.1371/journal.pone.0335199 (PMC12626332; doi:10.1371/journal.pone.0335199)
Supplement: Attachment — Submitted filename: Comment 1.docx [file pone.0335199.s002.docx]

Dear Editor,

I hope this message finds you well. I have completed my review of the manuscript entitled “Exploring Cardiovascular Disease Prevalence and Contributing Factors among Adults in Southern Iran, a Cross-Sectional Survey: Rationale, Design, and Primary Results” with manuscript Number: PONE-D-25-24170. Below, you will find my detailed comments and suggested revisions for your consideration.

Thank you for the opportunity to contribute to the peer review process.

Sincerely,

**Comment 1:**
Please revise the use of abbreviations throughout the manuscript. Ensure that each abbreviation is spelled out in full upon its first appearance in the text, followed by the abbreviation in parentheses.

**Comment 2:**
The manuscript contains several grammatical errors that affect the overall readability and clarity of the text. Additionally, there should be a space between the final word of a sentence and the subsequent reference citation. Please carefully revise the entire manuscript to address these issues and ensure proper formatting and grammatical accuracy throughout.

**Comment 3:**

While the authors mention using cluster and stratified random sampling, the explanation is vague and lacks key operational details. Please clarify how clusters were defined and selected, how stratification was applied, and whether urban/rural representation was proportionally allocated. A flow diagram or visual representation of the sampling strategy would also be beneficial.

**Comment 4:**

Although the use of WHO STEPS and GPAQ tools is noted, there is no mention of whether the questionnaire was pilot tested or validated in the local population. Please specify whether the adapted version underwent pretesting, and if so, how reliability and validity were assessed. This is crucial given cultural and linguistic differences that may affect data quality

**Comment 4:**

While the use of SPSS and basic tests is appropriate, further clarification is needed regarding **model model-building strategy** for multivariable logistic regression. For example, how were confounding variables selected? Was multicollinearity assessed? Also, please specify the criteria for inclusion/exclusion of variables in the final model and whether interaction terms were considered.

**Comment 5:**

Figures 1 and 2 are not properly indexed or cited within the main body of the manuscript. Currently, only the figure legends appear in the text, without any corresponding references or placements indicating where the figures should be consulted. Please ensure that both figures are explicitly referenced and appropriately positioned within the text to guide readers and enhance the clarity of the study’s methodology and results.

**Comment 6:**

The Discussion section does not adequately contextualize the study's findings in relation to previous research. A critical component of a well-structured discussion is the comparison and contrast of current results with those reported in existing literature. At present, this section lacks sufficient integration of relevant studies and does not effectively highlight consistencies or discrepancies with past findings. I recommend revising the Discussion to include a more comprehensive and analytical comparison with previous studies, thereby strengthening the interpretation and significance of the results.
