# Supplementary material for: Exploring cardiovascular disease prevalence and contributing factors among adults in Southern Iran, a cross-sectional survey: Rationale, design, and primary results
Source: PLoS One. 2025 Nov 18;20(11):e0335199. doi: 10.1371/journal.pone.0335199 (PMC12626332; doi:10.1371/journal.pone.0335199)
Supplement: Attachment — Submitted filename: CVD in Iran PLOS ONE is due by Jun 18 2025.docx [file pone.0335199.s003.docx]

This cross-sectional study by Farshdi et al investigates the **prevalence, risk factors, and management** of cardiovascular disease (CVD) in **Hormozgan Province, Iran.** The study includes data from over 6,500 males and females participants aged 20 and older using the WHO steps methodology with a high response rate (96.6%), increasing the validity and generalizability of findings to the local population. The authors highlight key sociodemographic, metabolic, behavioural, and psychological risk factors. While the study is valuable and timely, particularly in the context of growing noncommunicable disease burdens in the MENA region. However, some methodological and reporting issues need clarification to better understand the underlying the clinical relevance of these findings.

Major comments

The results of the analyses are interesting if somewhat predictable. Since it is not a longitudinal study, as a cross-sectional study, we can not interpret the association between risk factors and CVD. Authors need to clearly state this in the discussion and limitation section.

Table 5 shows the regression analysis of the variables such as depression, anxiety and son, but the definition and methods for the measurement of these variables are missing. Detailed methodology on how these variables were measured need to added with cut-offs or scoring system.

Univariate analysis show depression and anxiety are associated with CVD, however only severe anxiety remains significant in multivariate analysis. Again, authors need to explain how severity was categorized.

Table 5, smoking shows no association with CVD (OR = 1.038, p = 0.812), that contradicts with the literature or it could be likely self-reported bias and might not be fully reliable.

Minor Comments

Minor grammatical errors should be addressed to improve readability. All legends should explain all terms and abbreviations used.
