# Supplementary material for: Exploring cardiovascular disease prevalence and contributing factors among adults in Southern Iran, a cross-sectional survey: Rationale, design, and primary results
Source: PLoS One. 2025 Nov 18;20(11):e0335199. doi: 10.1371/journal.pone.0335199 (PMC12626332; doi:10.1371/journal.pone.0335199)
Supplement: Attachment — Submitted filename: Response to reviewers.1.docx [file pone.0335199.s005.docx]

**Response to reviewers**

**Dear Editor and Reviewers,**

We sincerely thank you for the time and effort invested in reviewing our manuscript. We greatly appreciate the insightful comments and constructive feedback provided, which have helped us to improve the quality of our case report. Below, we address each of the reviewer’s points in detail.

**Reviewer 1:**

The study was conducted among good numbers of samples with reasonable rate of response rate (Though little low for face-to-face but okay for online survey). Reliable tools (3 item UCLA loneliness Scale, and SAS_SV for PSU were utilized which are with good internal consistency. Some suggestions are:

1. **Avoid sub-headings in introduction section.**

Author response: We sincerely appreciate the reviewer’s thoughtful comment. We confirm that no sub-headings have been included in the Introduction section of the manuscript.

2. **Line 55, need to revise (Grammatical and other issue (i.e., stagma) need to be verified and redo.**
   Author response: We thank the reviewer for highlighting this potential issue. Based on the submitted PDF version, there is no text at line 55. To ensure we address your concern accurately, we revised the text through out to check grammar errors. Please let us know if we need to improve any other specific section.
3. **Line 135, No need to keep question here, just attach as annexure.**
    Author response: We sincerely appreciate the reviewer’s suggestion. However, we would like to respectfully clarify that the questionnaire is not included in the main text of our submitted manuscript. Moreover, according to the submitted PDF version, line 135 does not contain any reference to the questionnaire. We would be grateful if the reviewer could kindly provide further guidance or specify the exact section or text where the questionnaire is mentioned, so that we can address the concern appropriately.
4. **Line 193: Why total is kept in first row? I suggest to keep total in last row.**
   Author response: We thank the reviewer for this helpful suggestion. However, since the total population data are a key reference point in our analysis, we deliberately placed the “Total” row at the top of the table to highlight the overview of the data for readers.
5. **Line 210: There is no need to keep each tool's internal consistency score in description of results.**
   Author response: We are grateful for the reviewer’s attention to detail. According to the PDF we submitted, there is no mention of internal consistency scores at line 210. If this comment refers to another section, could you please advise us so we can make the appropriate revision? We are happy to address this promptly with your guidance.
6. **Chronbach alpha may be calculated again if the tool was modified in local language.**
   *Author response:* We truly appreciate this important point. The questionnaires used in our study had already undergone validation and reliability testing for the Iranian population in Persian. No further modifications were made in this study; therefore, recalculation of Cronbach’s alpha was not necessary.

We highly value your constructive feedback, which helps improve the clarity and quality of our manuscript. Should you require additional information or clarification on any point—especially regarding lines 55 and 210—please let us know.

**Reviewer 2:**

1. **In abstract include number of participants not sample size 6504...6298**

Author Response: Thank you for this suggestion. We have updated the abstract to clearly state the exact number of participants included in the study.

Page 2, lines 29

2. **In abstract result part: prevalence of abdominal obesity, DM, hypertension and success rate of treatment … Not needed; it will not go with the objectives of the study. Which BMI category, age, and sex were associated with CVD?**

Author Response: We sincerely thank the reviewer for their valuable comments, which have helped us improve the clarity and focus of our manuscript. In response, we have revised the Results section of the abstract by removing the prevalence data related to abdominal obesity, diabetes mellitus, hypertension, and treatment success rates. Instead, we have emphasized the associations between BMI, age, and sex with cardiovascular disease (CVD). however, both age and body mass index were analyzed as continuous variables. Our findings indicated that higher age and BMI are independently associated with an increased risk of CVD. Additionally, our analysis showed that females have a 33% lower risk of developing CVD compared to males (OR: 0.67; 95% CI: 0.53–0.85).

*Page 2, lines 40-44, Page 3, lines 47.*

The revised abstract results are as follows**: Results:** A total of 6,298 participants were included in the study, with a mean age of 45.63 ± 15.04 years. Among them, 58.5% were female, and 51.6% lived in urban areas. Key findings revealed that 43.9% of the population were identified as overweight or obese, with a significant prevalence of abdominal obesity at 69.6%, particularly among females. The prevalence of CVD in the population was 7.8%, with IHD affecting 6.7% of individuals and stroke affecting 2.2%. Multivariable logistic regression identified increased age (AOR: 1.03; 95% CI: 1.02–1.04), higher body mass index (BMI) (AOR: 1.02; 95% CI: 1.00–1.05), hypercholesterolemia (AOR: 1.30; 95% CI: 1.03–1.66), hypertension (HTN) (AOR: 2.02; 95% CI: 1.55–2.64), diabetes (AOR: 1.31; 95% CI: 1.00–1.71), and severe anxiety (AOR: 2.39; 95% CI: 1.30–4.39) as significant risk factors for CVD. Women had a 33% lower risk of having CVD compared to men (AOR: 0.67; 95% CI: 0.53–0.85).

3. **Line 111: remove "Participants aged 20 and older were recruited from design and geography…" as it was already in participant/population section**
   Author Response: Thank you for your constructive feedback, The redundant sentence has been removed for clarity and conciseness.
4. **Line 114/5: The study received approval from the Ethics Committee of Hormozgan University of Medical Sciences (IR.HUMS.REC.1401.080). Include it in the ethical part.**
   Author Response: We appreciate the reviewer’s comments. In accordance with your suggestion, the ethical approval information has been moved to the "Ethics Approval and Consent to Participate" section, after the Data Availability statement.

*Page 33, lines 549-551*

5. **Line 231 definition…>make it operational definition**
   Author Response: Thank you for your careful reading of our manuscript and for your helpful comments, Definitions have been revised and clarified as operational definitions.
6. **Line 234/35: … Medical documents were scanned and reviewed by a cardiologist to confirm the definitive diagnosis. For IHD. How ischemic stroke or hemorrhagic stroke was diagnosed was not explained.**
   Author Response: We are grateful for the reviewer’s thorough review and helpful suggestions, Additional details about the diagnostic criteria and confirmation process for ischemic and hemorrhagic stroke have been included to clarify this point.

*Page 14, lines 274-276*

7. **Line 260: At what p-value was statistical significance for all independent variables in the final model declared? How was the strength of association between independent and dependent variables assessed? Explain whether you used COR/AOR with a confidence interval.**
   Author Response: Thank you for your constructive feedback,We have specified that a p-value < 0.05 was used to define statistical significance. Additionally, we clarified that both Crude Odds Ratios (COR) and Adjusted Odds Ratios (AOR) with 95% confidence intervals were calculated to assess the strength of associations.

*Page 15, lines 299-306*

The revised Data analysis tools and methods section is as follows: All statistical analyses were conducted using SPSS version 26. Quantitative variables were described using means and standard deviations, while categorical and qualitative variables were represented by frequency counts and percentages. The Chi-square test was employed to compare the general characteristics of subjects between the two groups for qualitative variables. The Kolmogorov-Smirnov test was used to evaluate the normality of the data distribution. The Mann-Whitney test was applied to quantitative variables that did not exhibit a normal distribution. Univariate and multivariable logistic regressions were conducted to identify associations between various characteristics and metabolic variables with CVD. Variables that yielded a p-value less than 0.2 in the univariate analysis were included in the multivariable logistic regression model. In the multivariable model, we systematically removed variables with the highest p-values until all remaining variables had p-values below 0.05. Both crude odds ratios (COR) and adjusted odds ratios (AOR), along with their 95% confidence intervals (CIs), were calculated to quantify the strength of associations between independent variables and CVD. A p-value less than 0.05 was considered statistically significant.

8. **Line 358: prevalence of 7.8%....add confidence interval of prevalence**
   Author Response: The confidence interval has been added to the prevalence estimate as requested.

*Page 26, lines 410*

9. **Lines 368-375: There is no need to discuss smoking and hookah use, as there is no finding in this study.**
   Author response: Thanks for your comment, The discussion related to smoking and hookah use has been removed.
10. **Discussion and recommendation are mixed**
    Author Response: Thank you for your feedback on our manuscript. We made the changes accordingly. The discussion and recommendations have been separated into distinct sections for clarity.

*Page 27-29, lines 434-479*

11. **Discussion was not elaborated by comparing and contrasting findings with previous studies/literature**
    Author Response: Thank you for your feedback on our manuscript. We appreciate your comments, which will help improve our paper. As suggested by the esteemed reviewer, we have compared and discussed our study’s results in relation to findings from several relevant articles.

*Page 27-28, lines 434-460*

12. **Conclusion and recommendation were mixed. Conclusion was not narrating the research finding… focus on your findings**
    Author Response: Thank you for your comments ,The conclusion has been rewritten to focus clearly on the study’s key findings, with recommendations presented separately.

*Page 31-32, lines 529-533*

13. **No recommendation was given on isolated topic**
    Author Response: Specific recommendations related to isolated topics have now been added to the recommendation section.

*Page 29, lines 464-479*

**Reviewer 3:**

Reviewer #3: Dear Editor,
I hope this message finds you well. I have completed my review of the manuscript entitled “Exploring Cardiovascular Disease Prevalence and Contributing Factors among Adults in Southern Iran, a Cross-Sectional Survey: Rationale, Design, and Primary Results” with manuscript Number: PONE-D-25-24170. Below, you will find my detailed comments and suggested revisions for your consideration.
Thank you for the opportunity to contribute to the peer review process.
Sincerely,

Author response: Thank you very much for your thorough and constructive review of our manuscript. We appreciate the time and expertise you have invested in helping us improve our work. Below we address each of your comments in detail.

**Comment 1:
Please revise the use of abbreviations throughout the manuscript. Ensure that each abbreviation is spelled out in full at its first occurrence, followed by the abbreviated form in parentheses**.

Author response: We have carefully reviewed the manuscript and ensured that each abbreviation is spelled out in full at its first occurrence, followed by the abbreviation in parentheses. Subsequent use of abbreviations is consistent throughout the text.

**Comment 2:
The manuscript contains several grammatical errors that affect readability and clarity. Additionally, ensure there is a space between the final word of a sentence and the subsequent reference citation. Please revise the entire manuscript carefully to correct grammatical inaccuracies, improve clarity, and ensure consistent formatting throughout.**

Author response: We have thoroughly revised the manuscript to correct grammatical errors and improve overall readability and clarity. Additionally, we reviewed all reference citations to confirm appropriate spacing between the final word and the citation.

**Comment 3:
While the authors describe the use of cluster and stratified random sampling, the methodology lacks sufficient detail to assess its rigor. Please clarify:**

Author response: We greatly appreciate the reviewer’s pertinent observations regarding our sampling methodology. In response, we have revised and expanded the Methods section to provide greater clarity and detail.

**• Cluster Selection: How were clusters defined (e.g., geographic units, institutions)? What criteria were used for their selection?**

Author response: Clusters were defined as individual health service units (comprehensive health centers or health posts in urban areas; health service units in rural areas), and selected through simple random sampling from an updated registry.

*Page 8, lines 147-158*

**• Stratification: On what basis were strata established (e.g., socioeconomic status, disease prevalence)? How were sampling proportions determined?**

Author response: Stratification was conducted at two levels: first by county (geographic stratum), and then by urban/rural residence within each county, with sample allocation in each stage determined proportionally to the size of the adult population.

*Page 7-8, lines 144-168*

**• Urban/Rural Representation: Was the allocation proportional to population distribution? If not, justify the approach.**

Author response: Participant allocation between urban and rural areas within each county was strictly proportional to their respective population sizes.

Household selection within clusters was randomized and strictly followed a protocol to mitigate potential bias from non-responders.

*Page 7-8, lines 144-168*

**A flow diagram or visual summary of the sampling strategy would greatly enhance reproducibility and transparency.**

Author response: As recommended, a summary table outlining the complete sampling strategy has been included (see Table 1) to facilitate reproducibility and transparency. *Page 9, lines 170*

**Comment 4:
Although the use of WHO STEPS and GPAQ tools is noted, there is no mention of whether the questionnaire was pilot tested or validated in the local population. Please specify whether the adapted version underwent pretesting, and if so, how reliability and validity were assessed. This is crucial given cultural and linguistic differences that may affect data quality.**

Author response: We thank the reviewer for highlighting the importance of questionnaire validity and reliability in the study population. In response, we have clarified in the revised manuscript that The validity and reliability of the WHO STEPS instruments and other questionnaires, have been thoroughly evaluated in the Iranian context. All questionnaires employed in this study have previously been utilized in multiple rounds of the national Stepwise Approach to Noncommunicable Disease Risk Factor Surveillance (WHO STEPS) surveys in Iran, specifically in 2005, 2006, 2007, 2008, 2009, 2011, and 2016. Relevant references for these validation and reliability studies are provided in the Methods section of the manuscript to ensure transparency and facilitate further review.

*Page 10, lines 201-203*

**Comment 5:
While the use of SPSS and basic statistical tests is appropriate, the manuscript requires further clarification regarding the model-building strategy for the multivariable logistic regression. Specifically, please address the following:
• Variable Selection: How were confounding variables identified and selected for inclusion?
• Multicollinearity: Was multicollinearity assessed among predictors? If so, what measures were used (e.g., variance inflation factors, correlation matrices)?
• Inclusion/Exclusion Criteria: What statistical or theoretical criteria determined the final variables retained in the model (e.g., p-value thresholds, likelihood ratio tests)?
• Interaction Terms: Were potential interaction terms explored? If yes, how were they selected and evaluated? Providing these details will enhance the reproducibility and methodological rigor of the analysis.**

Author response: Thank you for your constructive feedback. We clarified the model-building strategy in the statistical analysis section as follows:

- **Variable Selection**: Potential confounders were identified based on prior literature and theoretical relevance, as well as initial univariate analyses with a p-value threshold of 0.20.
- **Multicollinearity**: We evaluated multicollinearity among the predictor variables by calculating Variance Inflation Factors (VIF). All VIF values were below 5, indicating that there was no significant multicollinearity affecting the model.
- **Inclusion/Exclusion Criteria**: In our study, the selection of variables for the multivariable logistic regression model was based on a combination of statistical and theoretical considerations. Initially, univariate logistic regression analyses were performed for each potential predictor variable to examine their individual associations with cardiovascular disease (CVD). Variables with a p-value less than 0.2 in the univariate analysis were considered for inclusion in the multivariable model. This more lenient threshold (p < 0.2) was chosen to avoid prematurely excluding potentially important variables. Subsequently, we employed a stepwise approach in the multivariable model: variables with the highest p-values were progressively removed one by one until all remaining variables demonstrated statistical significance at the p < 0.05 level. This ensured that the final model retained only those predictors independently associated with CVD. All analyses were conducted using SPSS version 26. Crude odds ratios (COR) and adjusted odds ratios (AOR) with their 95% confidence intervals (CI) were calculated to quantify association strengths. A significance level of p < 0.05 was used to determine statistical significance.
- **Interaction Terms**: In this study, we did not explore potential interaction terms. Our analysis focused on assessing the main effects of the selected variables based on prior literature and theoretical considerations.

**The revised Data analysis tools and methods section is as follows**: All statistical analyses were conducted using SPSS version 26. Quantitative variables were described using means and standard deviations, while categorical and qualitative variables were represented by frequency counts and percentages. The Chi-square test was employed to compare the general characteristics of subjects between the two groups for qualitative variables. The Kolmogorov-Smirnov test was used to evaluate the normality of the data distribution. The Mann-Whitney test was applied to quantitative variables that did not exhibit a normal distribution. Univariate and multivariable logistic regressions were conducted to identify associations between various characteristics and metabolic variables with CVD. Variables that yielded a p-value less than 0.2 in the univariate analysis were included in the multivariable logistic regression model. In the multivariable model, we systematically removed variables with the highest p-values until all remaining variables had p-values below 0.05. Both crude odds ratios (COR) and adjusted odds ratios (AOR), along with their 95% confidence intervals (CIs), were calculated to quantify the strength of associations between independent variables and CVD. A p-value less than 0.05 was considered statistically significant.

**Comment 6:
Figures 1 and 2 are not properly indexed or cited within the main body of the manuscript. Currently, only the figure legends appear in the text, without any corresponding references or placements indicating where the figures should be consulted. Please ensure that both figures are explicitly referenced and appropriately positioned within the text to guide readers and enhance the clarity of the study’s methodology and results.**

Author response: Thank you for your comment. We have updated the main text to explicitly reference Figures 1 and 2 at appropriate points within the Methods and Results sections. The figures are now properly numbered and positioned close to their citations to guide readers effectively.

*Page 6, lines 123, Page 23, lines 373*

**Comment 7:
The Discussion section does not adequately contextualize the study's findings in relation to previous research. A critical component of a well-structured discussion is the comparison and contrast of current results with those reported in existing literature. At present, this section lacks sufficient integration of relevant studies and does not effectively highlight consistencies or discrepancies with past findings. I recommend revising the Discussion to include a more comprehensive and analytical comparison with previous studies in your area, thereby strengthening the interpretation and significance of the results.**

Author response: Thank you for your careful reading of our manuscript and for your helpful comments. The Discussion has been substantially revised to better contextualize our findings within the broader literature. We added comparisons with relevant international studies, highlighting both consistencies and divergences.

*Page 27-29, lines 434-479*

**Reviewer #4:**

**No. the authors mention data restrictions. A thorough language revision by a native English speaker is recommended to enhance readability.

This cross-sectional study by Farshdi et al investigates the prevalence, risk factors, and management of cardiovascular disease (CVD) in Hormozgan Province, Iran. The study includes data from over 6,500 males and females participants aged 20 and older using the WHO steps methodology with a high response rate (96.6%), increasing the validity and generalizability of findings to the local population. The authors highlight key sociodemographic, metabolic, behavioural, and psychological risk factors. While the study is valuable and timely, particularly in the context of growing noncommunicable disease burdens in the MENA region. However, some methodological and reporting issues need clarification to better understand the underlying the clinical relevance of these findings.**

Author response: Thank you very much for your insightful and constructive review of our manuscript. We appreciate your recognition of the study’s value and the opportunity to improve the clarity and rigor of our work. We address your comments as follows:

**Major comments:**

**The results of the analyses are interesting if somewhat predictable. Since it is not a longitudinal study, as a cross-sectional study, we can not interpret the association between risk factors and CVD. Authors need to clearly state this in the discussion and limitation section**.

Author response: We agree that causal inference cannot be drawn from cross-sectional data. Accordingly, we have revised both the Discussion and Limitations sections to explicitly state that observed associations reflect correlation rather than causation, and that longitudinal studies are necessary to confirm temporal relationships.

*Page 28, lines 458-460, Page 30, lines 505-506*

**Table 5 shows the regression analysis of the variables such as depression, anxiety and son, but the definition and methods for the measurement of these variables are missing. Detailed methodology on how these variables were measured need to added with cut-offs or scoring system.**

Author response: We have now expanded the Methods section concerning the assessment of depression and anxiety to include a detailed description of the instruments used, the validated scales, scoring systems, and cut-off values applied to categorize severity levels.

*Page 11-12, lines 222-227*

**Univariate analysis show depression and anxiety are associated with CVD, however only severe anxiety remains significant in multivariate analysis. Again, authors need to explain how severity was categorized**.
Author response: We have clarified the categorization of anxiety severity in the Methods section by describing the criteria and cut-offs used to define mild, moderate, and severe anxiety based on the validated instrument scores.

*Page 11-12, lines 222-227*

**Table 5, smoking shows no association with CVD (OR = 1.038, p = 0.812), that contradicts with the literature or it could be likely self-reported bias and might not be fully reliable**.

Author response: We appreciate the reviewer’s valuable comment regarding the lack of association between smoking and cardiovascular disease (CVD) in our study. We acknowledge that this finding contrasts with much of the existing literature. Accordingly, we have revised the Discussion section to address this issue explicitly. In the updated text, we highlight the potential for self-report bias or underreporting of smoking status as possible reasons for the absence of a significant association. We also mention limitations related to capturing smoking intensity and duration, which may have contributed to this discrepancy.

*Page 28, lines 452-458*

**Minor Comments
Minor grammatical errors should be addressed to improve readability. All legends should explain all terms and abbreviations used.**

Author response: We have addressed minor grammatical errors throughout the manuscript with the assistance of a native English speaker to enhance overall readability.

All figure and table legends have been revised to fully explain terms and abbreviations to ensure clear understanding.
