# Supplementary material for: Exploring cardiovascular disease prevalence and contributing factors among adults in Southern Iran, a cross-sectional survey: Rationale, design, and primary results
Source: PLoS One. 2025 Nov 18;20(11):e0335199. doi: 10.1371/journal.pone.0335199 (PMC12626332; doi:10.1371/journal.pone.0335199)
Supplement: Attachment — Submitted filename: Response_to_reviewers.1_auresp_2.docx [file pone.0335199.s006.docx]

**Response to reviewer**

**Dear Editor and Reviewers,**

We sincerely appreciate the time and effort you have dedicated to reviewing our manuscript. Your insightful comments and constructive suggestions have been invaluable in enhancing the quality of our case report.

We have carefully considered each comment and have incorporated the necessary revisions into the manuscript.

Below, we provide detailed responses to the reviewers’ comments.

Thank you for your consideration.

**Reviewer #2:**

**1.** Line 408-410: Our study provides important insights into the CVD landscape in Hormozgan Province, revealing a prevalence of 7.8% (CI=0.071-0.084), with IHD accounting for 6.7% (0.044-0.09) and stroke for 2.2% (0.018-0.025)

1.1. Confidence intervals (CI) should be reported in the same unit as the main value. If you report prevalence as a percentage (e.g., 7.8%), then the CI should also be in percentage (CI: 7.1%–8.4%).

**Authors response:** Thank you for this helpful suggestion. We have revised the manuscript so that all confidence intervals are reported in the same units as the corresponding main values.

Page 24, line 391-392.

1.2. Discuss this finding (prevalence of CVD) by comparing and contrasting prevalence of CVD from other parts of the world. This helps the reader whether prevalence of CVD in the region is high or low compared to other parts of world.

**Authors response:** We sincerely thank the reviewer for their insightful and constructive comments. All the points requested have been thoroughly addressed and incorporated into the discussion section of the manuscript.

Page 24, line 392-395

**2.** Discussion line 445-451

Our logistic regression analysis …. increased age, increased BMI, hypercholesterolemia, diabetes mellitus, HTN, and severe anxiety…. include female sex

Hypertension and severe anxiety was discussed but increased age, increased BMI, hypercholesterolemia, diabetes mellitus and sex should be also discussed by comparing and contrasting with previous studies (whether your finding is similar or different)

**Authors response:** We are grateful for the reviewer’s attention to detail. Age, BMI, hypercholesterolemia, diabetes mellitus, and sex were discussed by comparing and contrasting our findings with those of previous studies.

Page 25-26, line 420-442

**3.** Line 463-465 and 471-472: Our study further emphasizes tailoring interventions, particularly to increase physical activity among women and urban populations….no finding in your study that suggest physical activity in women and urban population associated to CVD. Remove it

**Authors response:** Thank you for your constructive feedback. Following the reviewer’s suggestions, we have removed the items referenced in the comments and revised the manuscript accordingly.

Page 27, line 462-463 and line 455-456

**4.** Line 469-70: even though prevalence of obesity is high, no finding in your study that suggest obesity associated factor to CVD: better to remove it.

**Authors response:** Thank you for this helpful suggestion. Following the reviewer’s suggestions, we have removed the items referenced in the comments and revised the manuscript accordingly.

Page 27, line 461

**5.** Conclusion

Focus on your objectives

example like this. The prevalence of CVD was 7.8%(is it high/low comparing…). Factors significantly associated with CVD included older age, higher BMI, hypercholesterolemia, diabetes mellitus, hypertension, and severe anxiety. Interestingly, female sex appeared to be protective against CVD compared to male sex.

**Authors response:** We sincerely thank you for your constructive feedback, which has been invaluable in improving the quality of our article. We have implemented the referee’s suggestions and corrected the conclusion.

Page 28, line 486-489
